# Burnout and Associated Risk Factors in Pediatric Residents

**DOI:** 10.31486/toj.20.0037

**Published:** 2021

**Authors:** Abdulmajeed Bin Dahmash, Mohammed Faisal Alajmi, Abdulrahman Yousef Aldayel, Yasir Tariq Alotaibi, Sultan Meshal Altoum, Abdullah Alzayed, Moslah Ali Jabari

**Affiliations:** Department of Pediatrics, College of Medicine, Imam Mohammad ibn Saud Islamic University, Riyadh, Saudi Arabia

**Keywords:** *Burnout–professional*, *burnout–psychological*, *internship and residency*, *pediatrics*, *work-life balance*, *workplace*

## Abstract

**Background:** Burnout is a syndrome characterized by emotional exhaustion, depersonalization, and a low sense of personal accomplishment. The aim of this study was to identify burnout incidence in pediatric residents and evaluate possible risk factors for burnout.

**Methods:** Using a cross-sectional study design, we approached all pediatric residents in the Saudi Pediatrics Residency Program in Riyadh, Saudi Arabia (n=457) between January and March 2019. The Maslach Burnout Inventory-Human Services Survey was used to assess burnout incidence. In addition, demographic factors, schedule burden, career choice satisfaction, and work-life balance were assessed.

**Results:** The response rate was 57.8% (264/457). Males represented 46.6%. Only 14% of the residents in the study were satisfied with their work-life balance, and 62% were satisfied with their career choice of pediatrics. The overall high burnout incidence was 15.9%, the high emotional exhaustion incidence was 63.6%, the high depersonalization incidence was 27.7%, and the low sense of personal accomplishment incidence was 48.5%. In the multivariate analysis, an increase in the average number of on-calls per month (odds ratio [OR]=1.66, 95% CI 1.12-2.46; *P*=0.012) and satisfaction with salary (OR=0.47, 95% CI 0.33-0.66; *P*<0.001) showed significant associations with high overall burnout.

**Conclusion:** We found a high level of emotional exhaustion and a low sense of personal accomplishment among respondents. However, less than one-third of residents had feelings of depersonalization or overall high burnout. Residency program directors may need to make modifications in their programs to ensure a good work-life balance for residents that will help ensure that these physicians provide safe and sustained patient care.

## INTRODUCTION

Practicing medicine while studying can be challenging, and studies have shown that the incidence of burnout among pediatric residents ranges from 17% to 67.8%.^1^ Burnout is a syndrome characterized by emotional exhaustion, depersonalization, and a low sense of personal accomplishment.^2^ Symptoms include anger, blame, helplessness, hopelessness, treating patients as impersonal objects, loneliness, and frustration with patients and with the health care system.^3^ Overburdened schedules, increased on-call scheduling, lack of mentorship, an unsupportive family or partner, dissatisfaction with work-life balance, and a lack of self-care are all risk factors that can precipitate burnout.^4-6^

Physicians with burnout are in danger of making medical errors and have lower patient satisfaction rates than non–burned-out physicians.^7,8^ While burnout has been investigated in many specialties, burnout among pediatric residents in Saudi Arabia has been recognized only recently (2018).^9^ A single-center study of 32 pediatric residents in Jeddah, Saudi Arabia, showed high emotional exhaustion in 43% of participants.^9^ Understanding burnout incidence and the risk factors among residents can help residency program directors control the working environment to create a support system that can reduce burnout incidence.^6^

Residency is a stressful phase in a physician's career and an ideal time to implement personal and organizational plans to decrease burnout incidence.^10^ We investigated burnout among pediatric residents in Saudi Arabia using a validated measure. We also evaluated possible risk factors, including demographic factors, schedule burden, lack of career choice satisfaction, and work-life balance.

## METHODS

Institutional review board approval was obtained from the College of Medicine, Imam Mohammad ibn Saud Islamic University, for this quantitative cross-sectional study. We distributed a survey to all pediatric residents in the city of Riyadh (n=457) between January and March 2019. The survey was distributed to residents during their academic activities at each center and was collected at the end of the activities. The first page of the survey included a description of the study and a consent form. We did not ask for the name of the resident or any detail that could reveal the resident's identity. Participation was totally voluntary.

The first part of the survey collected demographic information (age, sex, marital status, and residency level/postgraduate year), health-related factors (smoking status, hours of sleep/day, exercise days/week), and work-related factors (average on-calls/month, average working hours/day, number of clinics/week, and average number of patients under daily care). Satisfaction with work-life balance, choice of pediatrics as a career, and salary was measured using a 5-point Likert-type scale ranging from 1 (“very satisfied”) to 5 (“very unsatisfied”). Prior to the analysis, scores were recoded so that 5 corresponded to very satisfied and 1 corresponded to very unsatisfied. The second part of the survey was the Maslach Burnout Inventory-Human Services Survey (MBI-HSS), a validated measure of burnout.^11^

The MBI-HSS includes 22 questions that evaluate 3 domains of burnout: emotional exhaustion (9 questions), depersonalization (5 questions), and sense of personal accomplishment (8 questions). The severity of each item is assessed on a 7-point Likert scale, ranging from 0 (“never”) to 6 (“always”). Higher emotional exhaustion and depersonalization scores are associated with higher levels of burnout, whereas higher personal accomplishment scores are associated with lower levels of burnout. High emotional exhaustion was defined as a score >26, and high depersonalization was defined as a score >12. Low sense of personal accomplishment was defined as a score <32. A high risk of burnout was defined as coexisting high emotional exhaustion, high depersonalization, and low sense of personal accomplishment.

Statistical analysis was performed using SPSS, version 24.0 (IBM Corp). Counts and percentages were used to summarize participant demographics. Mean ± SD was used to summarize continuous variables. Burnout dimensions and overall burnout were analyzed as continuous and categorical (dichotomous) variables based on the previously mentioned cutoff criteria. Likert-scale items—satisfaction with work-life balance, career choice, and salary—were summarized using counts and percentages. Binary logistic regression was used to assess the predictors of the burnout dimensions as well as overall burnout. Univariate binary logistic regression was used to assess the associations of demographics and residency-related factors with burnout. Four models were constructed, 1 for each of the 3 dimensions of burnout and 1 for overall burnout. In addition, a chi-square test of independence was used to assess whether the distribution of each dependent variable across the various demographics was significantly different from what was expected under the null hypothesis. Multivariate binary logistic regression was used to assess the independent predictors of high risk of burnout. All independent variables were initially included in the model. A backward approach (using the *P* value for the Wald chi-square statistic) was used for variable selection. Variables were kept in the model if the *P* value for the multivariate Wald chi-square statistic in the final model was <0.1. Two-tailed hypothesis testing was performed at α≤0.05. Odds ratios (ORs) and 95% CIs were calculated for the independent variables included in the analysis. Exercise, age, and residency level were dichotomized prior to the analysis. The dichotomized versions of the burnout dimensions were handled as dependent variables. Satisfaction levels with work-related aspects were treated as continuous variables. Model performance was assessed by using *R*^2^ (the proportion of variance explained by the independent variables).

## RESULTS

Of the 457 surveys distributed, 264 surveys were returned for a response rate of 57.8%. Demographic characteristics of the respondents are shown in Table 1. Satisfaction with work-life balance, career, and salary is shown in the Figure.

**Figure 1.**
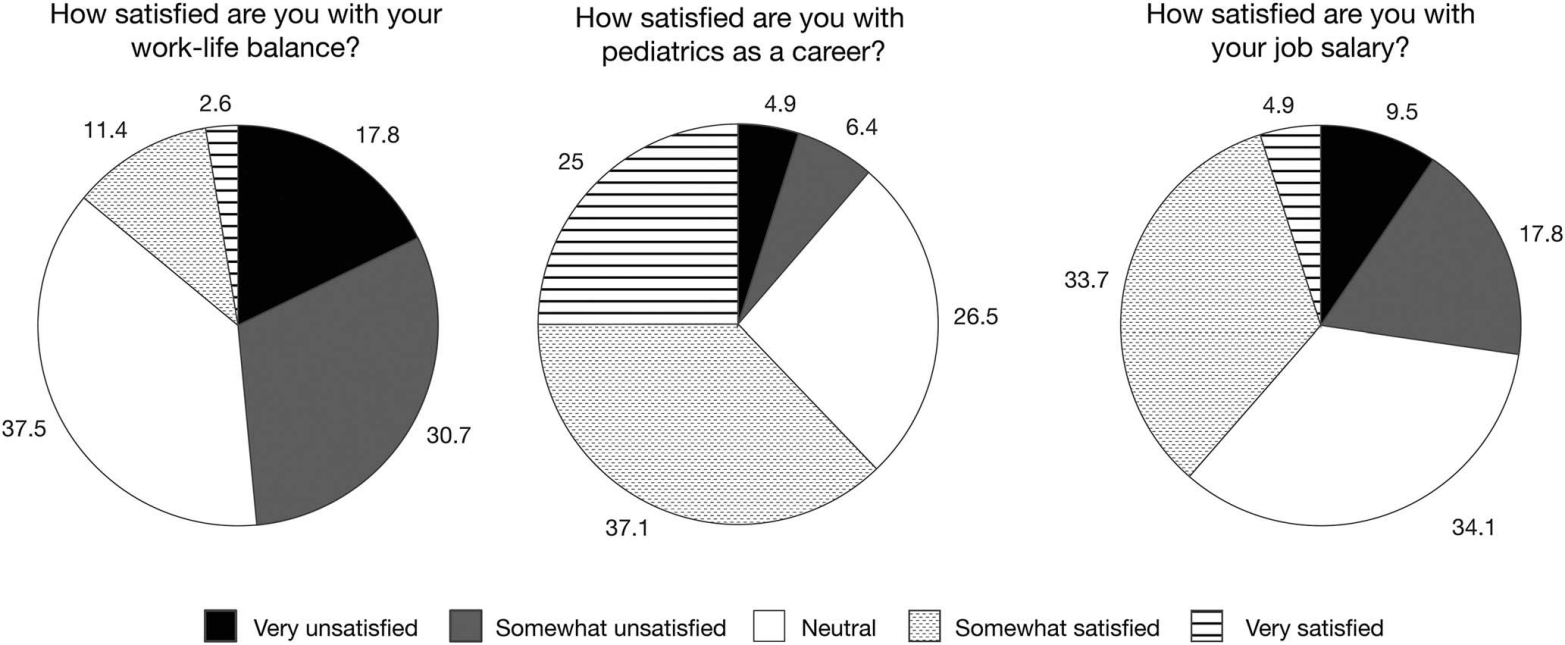
Respondents’ satisfaction with work-life balance, career choice of pediatrics, and salary.

**Table 1.** Demographic Characteristics of the Pediatric Resident Respondents (n=264)

Variable	Value
Age, years, n (%)	
<25	7 (2.7)
25-27	173 (65.5)
28-30	79 (29.9)
31-33	3 (1.1)
34-36	2 (0.8)
Sex, n (%)	
Female	141 (53.4)
Male	123 (46.6)
Marital status, n (%)	
Unmarried	173 (65.5)
Married	91 (34.5)
Residency year, n (%)	
1	108 (40.9)
2	75 (28.4)
3	45 (17.0)
4	36 (13.6)
Smoking status, n (%)	
No	198 (75.0)
Yes	66 (25.0)
Exercise, n (%)	
Never	139 (52.7)
≥1 day/week	125 (47.3)
Hours of sleep/day, mean ± SD	6.15 ± 1.33
Number of on-calls/month, mean ± SD	5.16 ± 0.91
Number of hours working in the hospital/day, mean ± SD	8.81 ± 2.31
Number of clinics/week, mean ± SD	2.29 ± 2.95
Number of patients under daily care, mean ± SD	5.63 ± 3.71

High emotional exhaustion was reported by 63.6% of residents (n=168), while a low sense of personal accomplishment was expressed by 48.5% (n=128). High depersonalization was the least prevalent of all burnout dimensions 27.7% (n=73). High risk of overall burnout was present in 15.9% (n=42) of the pediatric residents in this study (Table 2).

**Table 2.** Mean Scores for Burnout Domains, Prevalence of Negative Scores, and Prevalence of Overall High Burnout Among Pediatric Residents (n=264)

Variable	Value
Continuous presentation, mean ± SD (range)	
Emotional exhaustion	30.8 ± 11.6 (2-54)
Depersonalization	9.51 ± 5.91 (1-26)
Sense of personal accomplishment	30.9 ± 8.45 (8-47)
Categorical presentation, n (%)	
High emotional exhaustion	
No	96 (36.4)
Yes	168 (63.6)
High depersonalization	
No	191 (72.3)
Yes	73 (27.7)
Low personal accomplishment	
No	136 (51.5)
Yes	128 (48.5)
High burnout	
No	222 (84.1)
Yes	42 (15.9)

Notes: High emotional exhaustion was defined as a score >26, high depersonalization was defined as a score >12, and low sense of personal accomplishment was defined as a score <32. High burnout was defined as coexisting high emotional exhaustion, high depersonalization, and low personal accomplishment.

Univariate logistic regression analysis (data not shown) showed that having more on-calls/month (OR=1.58, 95% CI 1.17-2.12; *P*=0.003) and more patients under daily care (OR=1.09, 95% CI 1.01-1.19; *P*=0.035) were both significantly associated with high emotional exhaustion. The probability of high emotional exhaustion increased by 58% for each additional on-call in the month. Similarly, the probability of high emotional exhaustion increased by 9% for each additional patient under the daily care of a pediatric resident in this study. None of the remaining work-related variables showed a statistically significant association with emotional exhaustion.

High depersonalization was associated significantly only with the average number of on-calls/month (OR=1.57, 95% CI 1.14-2.15; *P*=0.005) (data not shown). None of the remaining work-related variables showed a statistically significant association with depersonalization. None of the work-related variables showed a statistically significant association with low sense of personal accomplishment.

Table 3 shows the association of demographic and work-related variables with high overall burnout. More on-calls/month was significantly associated with the risk of high burnout (OR=1.88, 95% CI 1.27-2.77; *P*=0.001). The probability of burnout increased by 88% for each increase in on-calls/month. None of the remaining variables was significantly associated with high risk of burnout.

**Table 3.** Association of Demographic and Work-Related Factors With High Burnout

Variable	High Burnout n=42	Odds Ratio (95% CI)	*P* Value (LR)^a^	*P* Value^b^
Age, years, n (%)				0.501
<28	31 (73.8)	Reference	Reference	
≥28	11 (26.2)	0.73 (0.33-1.50)	0.403	
Sex, n (%)				0.982
Female	23 (54.8)	Reference	Reference	
Male	19 (45.2)	0.94 (0.48-1.83)	0.852	
Marital status, n (%)				0.994
Unmarried	27 (64.3)	Reference	Reference	
Married	15 (35.7)	1.07 (0.52-2.12)	0.846	
Residency year, n (%)				
1 and 2	33 (78.6)	Reference	Reference	0.217
3 and 4	9 (21.4)	0.58 (0.25-1.23)	0.158	
Smoking status, n (%)				1.000
No	31 (73.8)	Reference	Reference	
Yes	11 (26.2)	1.09 (0.49-2.26)	0.833	
Exercise, n (%)				0.640
Never	24 (57.1)	Reference	Reference	
≥1 days/week	18 (42.9)	0.81 (0.41-1.57)	0.532	
Hours of sleep/day, mean ± SD	6.24 ± 1.51	1.06 (0.83-1.35)	0.646	0.681
Number of on-calls/month, mean ± SD	5.57 ± 0.99	1.88 (1.27-2.77)	0.001	**0.004**
Number of hours working in the hospital/day, mean ± SD	8.79 ± 0.84	1.00 (0.86-1.15)	0.948	0.906
Number of clinics/week, mean ± SD	2.05 ± 2.88	0.97 (0.86-1.09)	0.565	0.560
Number of patients under daily care, mean ± SD	6.17 (3.63)	1.04 (0.96-1.13)	0.314	0.305

Note: High burnout was defined as coexisting high emotional exhaustion, high depersonalization, and low personal accomplishment.

^a^Univariate logistic regression (LR).

^b^Chi-square test (categorical variables) or *t* test (continuous variables).

As shown in Table 4, high satisfaction with job salary was associated with lower odds of high emotional exhaustion (OR=0.50, 95% CI 0.38-0.67; *P*<0.001). For each 1-unit increase in satisfaction with job salary, the odds of high emotional exhaustion decreased by 50%. Similarly, for each 1-unit increase in satisfaction with job salary, the odds of high depersonalization also decreased by approximately 50% (OR=0.52, 95% CI 0.39-0.68; *P*<0.001). A 1-unit increase in job satisfaction score was associated with a 32% decrease in the odds of low sense of personal accomplishment (OR=0.68, 95% CI 0.53-0.87; *P*=0.002). Overall, the odds of high burnout decreased by 56% for each 1-unit increase in satisfaction with job salary (OR=0.44, 95% CI 0.32-0.62; *P*<0.001).

**Table 4.** Univariate Logistic Regression Analysis of Career Variable Satisfaction and Burnout Domains and High Burnout

	High Emotional		High		Low Personal		High	
	Exhaustion		Depersonalization		Accomplishment		Burnout	
Career Variable	OR (95% CI)	*P* Value	OR (95% CI)	*P* Value	OR (95% CI)	*P* Value	OR (95% CI)	*P* Value
Salary	0.50 (0.38-0.67)	<0.001	0.52 (0.39-0.68)	<0.001	0.68 (0.53-0.87)	0.002	0.44 (0.32-0.62)	<0.001
Pediatrics career	0.47 (0.35-0.64)	<0.001	0.71 (0.55-0.92)	0.008	0.55 (0.43-0.71)	<0.001	0.59 (0.44-0.79)	0.001
Work-life balance	0.28 (0.20-0.41)	<0.001	0.48 (0.35-0.65)	<0.001	0.70 (0.55-0.90)	0.006	0.42 (0.29-0.63)	<0.001

Notes: Odds ratios (ORs) and 95% CIs represent each 1-unit increase in satisfaction for the career variables. High burnout was defined as coexisting high emotional exhaustion, high depersonalization, and low personal accomplishment.

Each 1-unit increase in satisfaction with the choice of pediatrics as a career was associated with a 53% decrease in the odds of high emotional exhaustion (OR=0.47, 95% CI 0.35-0.64; *P*<0.001), a 29% decrease in the odds of high depersonalization (OR=0.71, 95% CI 0.55-0.92; *P*=0.008), a 45% decrease in the odds of low sense of personal accomplishment (OR=0.55, 95% CI 0.43-0.71; *P*<0.001), and an overall 41% decrease in the odds of high burnout (OR=0.59, 95% CI 0.44-0.79; *P*=0.001).

Each 1-unit increase in satisfaction with work-life balance was significantly associated with lower likelihood of high emotional exhaustion (*P*<0.001), high depersonalization (*P*<0.001), low sense of personal accomplishment (*P*=0.006), and high burnout (*P*<0.001).

As displayed in Table 5, male respondents were at lower risk of high emotional exhaustion than females (OR=0.51, 95% CI 0.29-0.99; *P*=0.025), and the risk of high emotional exhaustion was 2.5 times higher in smokers compared to nonsmokers (OR=2.49, 95% CI, 1.12-5.53; *P*=0.046). Each 1-call increase in the average number of on-calls/month was associated with a 59% increase in the odds of high emotional exhaustion (OR=1.59; 95% CI, 1.09-2.31, *P*=0.015). Each 1-unit increase in satisfaction with salary was associated with a 38% decrease in the odds of high emotional exhaustion (OR=0.62, 95% CI 0.44-0.88; *P*=0.007). Each 1-unit increase in satisfaction with work-life balance was associated with a 66% decrease in the odds of high emotional exhaustion (OR=0.34, 95% CI 0.23-0.49; *P*<0.001).

**Table 5.** Multivariate Logistic Regression Analysis Results

	High Emotional		High		Low Personal		High	
	Exhaustion		Depersonalization		Accomplishment		Burnout	
Variable	OR (95% CI)	*P* Value	OR (95% CI)	*P* Value	OR (95% CI)	*P* Value	OR (95% CI)	*P* Value
Sex								
Male	0.51 (0.29-0.99)	0.025						
Smoking status								
Yes	2.49 (1.12-5.53)	0.046						
Residency year								
3 and 4			0.49 (0.25-0.97)	0.04				
Number of on-calls/month	1.59 (1.09-2.31)	0.015					1.66 (1.12-2.46)	0.012
Salary satisfaction	0.62 (0.44-0.88)	0.007	0.61 (0.45-0.82)	0.001			0.47 (0.33-0.66)	<0.001
Pediatrics career satisfaction					0.55 (0.43-0.71)	<0.001		
Work-life balance satisfaction	0.34 (0.23-0.49)	<0.001	0.57 (0.41-0.8)	0.001				
*R*^2^	37.7%		13.9%		12%		19.1%	

Notes: Only variables included in the final model using stepwise backward elimination are displayed. High burnout was defined as coexisting high emotional exhaustion, high depersonalization, and low personal accomplishment.

OR, odds ratio; *R*^2^, coefficient of determination or the proportion of variance in the dependent variable that is explained by the model.

Year 3 and 4 residents were at lower risk of high depersonalization than year 1 and 2 residents (OR=0.49, 95% CI 0.25-0.97; *P*=0.04). Each 1-unit increase in satisfaction with job salary was associated with a 39% decrease in the odds of high depersonalization (OR=0.61, 95% CI 0.45-0.82; *P*=0.001). Similarly, each 1-unit increase in satisfaction with work-life balance was associated with a 43% decrease in the odds of high depersonalization (OR=0.57, 95% CI 0.41-0.80; *P*=0.001).

Each 1-unit increase in satisfaction with career choice of pediatrics was associated with a 45% reduction in the odds of low sense of personal accomplishment (OR=0.55, 95% CI 0.43-0.71; *P*<0.001).

Each 1-call increase in the average number of on-calls/month was associated with a 66% increase in the odds of high burnout (OR=1.66, 95% CI 1.12-2.46; *P*=0.012), and each 1-unit increase in satisfaction with salary was associated with a 53% reduction in the odds of high burnout (OR=0.47, 95% CI 0.33-0.66; *P*<0.001).

## DISCUSSION

Burnout—a constellation of emotional exhaustion, a state of depersonalization, and a diminished feeling of achievement—is caused by interactions between environmental stressors and genetic tendencies.^5^ Burnout can lead to physical and psychological symptoms, as well as substance abuse. All of these factors can negatively affect the life quality of residents and impact their ability to provide competent and safe patient care while meeting their responsibilities to study and learn.^5^ The Accreditation Council for Graduate Medical Education has stated that general practice residents, a group that includes pediatric residents, are among the medical education learners most affected by burnout.^12^

The prevalence of emotional exhaustion and low sense of personal accomplishment among the residents sampled for our study was 63.6% and 48.5%, respectively. These findings are similar to those of previous studies.^13-15^ Depersonalization was the least prevalent of the 3 dimensions of burnout. The incidence of high burnout was 15.9% among the respondents. In a large review, McKinley et al showed that the prevalence of burnout varies between 17% and 67.8% among pediatric residents.^1^ Pantaleoni et al found that burnout increases from 17% to 46% between the start of residency and the mid-intern level.^16^ However, in our multivariate regression analyses (Table 5), we found no significant differences between the year 1 and 2 residents and year 3 and 4 residents in prevalence of high burnout. The number of on-calls showed a significant association with high burnout. In a study of medical and surgical residents at 3 hospitals in Saudi Arabia, Hameed et al found that increased working hours/week was significantly associated with burnout.^17^ Kassam et al studied resident physicians at the University of Calgary in Canada to assess predictors of well-being and demonstrated that personal burnout and work dissatisfaction were the only significant indicators of overall well-being.^18^ However, on-calls affected residents’ sleep quality and minimized their leisure and family time.

Study limitations include the use of a survey that creates a dependence on self-reported data at risk for bias. Moreover, data were collected from one city only. An additional limitation of this study is the high proportion of first- and second-year residents relative to the higher levels. The Saudi Commission for Health Specialties (SCFHS)—which is responsible for all residency programs in Saudi Arabia—significantly increased the number of residents accepted in 2017 and 2018, and that increase may be the reason for the higher number of year 1 and 2 residents vs year 3 and 4 residents included in this study. Another limitation is that residents who experienced preexisting anxiety, depression, or other mood-related disorders were not excluded from participation. Also, as with all cross-sectional studies, this study offers relationships but is unable to posit causation. Thus, prospective cohort studies should be conducted to verify the outcomes reported.

An advantage of this study is its sample size that reflects a representative cross-section of pediatric residents in the capital city of Saudi Arabia. Although survey respondents were residents from different pediatric residency programs in Riyadh, Saudi Arabia, we did not intend to compare the results from each residency program. This study illustrates the incidence of burnout in pediatric residents overall, and SCFHS can potentially use this understanding of burnout among pediatric residents to create standardized organizational plans for all residency programs to improve residents’ well-being.

## CONCLUSION

Saudi pediatric residents reported high levels of emotional exhaustion and a low sense of personal accomplishment, although less than one-third of residents had feelings of depersonalization or overall high burnout. These findings suggest that residency program directors need to make adjustments to ensure a better work-life balance for residents, which would promote safe and sustained patient care. Prospective studies involving a large cohort of residents from different medical and surgical specialties and from a variety of regions are needed to reproduce these outcomes and to identify other factors that drive burnout.

## Notes

*This article meets the Accreditation Council for Graduate Medical Education and the American Board of Medical Specialties Maintenance of Certification competencies for Medical Knowledge, Professionalism, and Systems-Based Practice.*

## References

[1] McKinley TF, Boland KA, Mahan JD. Burnout and interventions in pediatric residency: a literature review. Burn Res. 2017;6:9-17. doi: 10.1016/j.burn.2017.02.003

[2] Maslach C, Jackson SE, Leiter MP. Maslach Burnout Inventory. 3rd ed. Consulting Psychologists Press; 1996.

[3] Meier DE, Back AL, Morrison RS. The inner life of physicians and care of the seriously ill. JAMA. 2001;286(23):3007-3014. doi: 10.1001/jama.286.23.3007 11743845

[4] Bin Dahmash A, Alorfi FK, Alharbi A, Aldayel A, Kamel AM, Almoaiqel M. Burnout phenomenon and its predictors in radiology residents. Acad Radiol. 2020;27(7):1033-1039. doi: 10.1016/j.acra.2019.09.024 31629625

[5] Ishak WW, Lederer S, Mandili C, Burnout during residency training: a literature review. J Grad Med Educ. 2009;1(2):236-242. doi: 10.4300/JGME-D-09-00054.1 21975985 PMC2931238

[6] Spickard A Jr, Gabbe SG, Christensen JF. Mid-career burnout in generalist and specialist physicians. JAMA. 2002;288(12):1447-1450. doi: 10.1001/jama.288.12.1447 12243624

[7] Wright AA, Katz IT. Beyond burnout - redesigning care to restore meaning and sanity for physicians. N Engl J Med. 2018;378(4):309-311. doi: 10.1056/NEJMp1716845 29365301

[8] Panagioti M, Panagopoulou E, Bower P, Controlled interventions to reduce burnout in physicians: a systematic review and meta-analysis. JAMA Intern Med. 2017;177(2):195-205. doi: 10.1001/jamainternmed.2016.7674 27918798

[9] Jamjoom RS, Park YS. Assessment of pediatric residents burnout in a tertiary academic centre. Saudi Med J. 2018;39(3):296-300. 29543309 10.15537/smj.2018.3.22328 PMC5893920

[10] West CP, Dyrbye LN, Erwin PJ, Shanafelt TD. Interventions to prevent and reduce physician burnout: a systematic review and meta-analysis. Lancet. 2016;388(10057):2272-2281. doi: 10.1016/S0140-6736(16)31279-X 27692469

[11] Maslach C, Jackson SE. The measurement of experienced burnout. J Organ Behav. 1981;2(2):99-113. doi: 10.1002/job.4030020205

[12] Accreditation Council for Graduate Medical Education. Committee CLER: Pathways to Excellence. 2013:204-205.

[13] Bin Dahmash A, Alhadlaq AS, Alhujayri AK, Alkholaiwi F, Alosaimi NA. Emotional intelligence and burnout in plastic surgery residents: is there a relationship? Plast Reconstr Surg Glob Open. 2019;7(5):e2057. doi: 10.1097/GOX.0000000000002057 31333920 PMC6571340

[14] Peterlini M, Tibério IFLC, Saadeh A, Pereira JCR, Martins MA. Anxiety and depression in the first year of medical residency training. Med Educ. 2002;36(1):66-72. doi: 10.1046/j.1365-2923.2002.01104.x 11849526

[15] Yaghmour NA, Brigham TP, Richter T, Causes of death of residents in ACGME-accredited programs 2000 through 2014: implications for the learning environment. Acad Med. 2017;92(7):976-983. doi: 10.1097/ACM.0000000000001736 28514230 PMC5483979

[16] Pantaleoni JL, Augustine EM, Sourkes BM, Bachrach LK. Burnout in pediatric residents over a 2-year period: a longitudinal study. Acad Pediatr. 2014;14(2):167-172. doi: 10.1016/j.acap.2013.12.001 24602580

[17] Hameed TK, Masuadi E, Al Asmary NA, Al-Anzi FG, Al Dubayee MS. A study of resident duty hours and burnout in a sample of Saudi residents. BMC Med Educ. 2018;18(1):180. doi: 10.1186/s12909-018-1300-5 30071835 PMC6090868

[18] Kassam A, Horton J, Shoimer I, Patten S. Predictors of well-being in resident physicians: a descriptive and psychometric study. J Grad Med Educ. 2015;7(1):70-74. doi: 10.4300/JGME-D-14-00022.1 26217426 PMC4507932

